# Solid breast tumors phantom emulating oxy and deoxyhemoglobin concentrations for near infrared imaging

**DOI:** 10.1371/journal.pone.0325768

**Published:** 2025-06-20

**Authors:** María Victoria Waks-Serra, Demián Augusto Vera, Nicolás Abel Carbone, Héctor Alfredo García, Pamela Alejandra Pardini, Juan Antonio Pomarico, Daniela Inés Iriarte

**Affiliations:** 1 CIFICEN (UNCPBA-CICPBA-CONICET), Tandil, Buenos Aires, Argentina; 2 BIONIRS Arg SA, Tandil, Buenos Aires, Argentina; Brandeis University, UNITED STATES OF AMERICA

## Abstract

**Significance:**

Continuous-wave near-infrared spectroscopy (CW-NIRS) is a valuable, inexpensive and non-invasive tool for complementary diagnose breast cancer. The use of phantoms has proven to be a very powerful way to evaluate different experimental approaches as well as to test possible diagnostics equipment. The phantoms developed in this work can properly emulate either benign or malignant tumors and, in contrast to those constructed with actual biological chromophores, require no special storage, being thus stable in time.

**Aim:**

In this work we study the feasibility of employing two artificial absorbents as a replacement for oxygenated and deoxygenated hemoglobin concentrations in breast tumors, allowing discrimination benign from malignant tumors in CW transmittance NIRS experiments.

**Approach:**

Tumor phantoms were made of epoxy resin containing two kinds of absorbents to emulate the absorption curves of the hemoglobins in concentrations that reproduce those of benign and malignant tumors (fibroadenoma and adenocarcinoma respectively). CW transmittance NIRS experiments were carried out to evaluate the approach which was also compared with Monte Carlo (MC) simulations.

**Results:**

Results show that the constructed tumor phantoms are feasible to reproduce the desired targets. Additionally, the retrieved concentrations agree with the proposed ones. Thus, it is possible to construct a phantom containing inclusions emulating a fibroadenoma and/or an adenocarcinoma suitable for testing mammography algorithms or equipment.

**Conclusions:**

We have successfully designed, constructed, and validated tumor phantoms emulating both benign and malignant breast tumors. The transmittance experiments carried out agree very well with MC simulations. In addition, it was possible to obtain a map of absorbent concentrations recovered from the diffuse imaging experiments.

## 1 Introduction

The development of diagnostic systems and medical therapies based on light requires the employment of phantoms as standard calibrated samples for testing and validation [1–6] These phantoms are tissue-simulating objects designed to mimic the properties of biological tissues. However, the elaboration of phantoms that correctly reproduce the optical characteristics of the principal components of biological tissue has been a significant challenge for researchers in biomedical areas [7–9]. In particular, diffuse optical imaging by near-infrared spectroscopy (NIRS) uses light in the wavelength range between 600 nm and 1000 nm (the so-called *optical window*) to image tissue. The procedure in turn allows for the reconstruction of maps of the concentrations of major tissue chromophores such as oxy and deoxyhemoglobin (HbO and HbR, respectively) [10–13]. These hemoglobin species are considered important markers for the monitoring and detection of diseases that require metabolic information for their diagnosis such as breast cancer [4, 8, 14, 15]. Moreover, the relative concentrations of HbO and HbR determine whether a lesion is either benign or malignant [14, 16–19]. As stated in previous reports, high levels of hemoglobin concentration in confined areas are characteristic of the presence of tumors when compared to the surrounding healthy tissue within the same breast [14, 15, 17, 20]. These studies indicate that the carcinogenic effects induced by changes in tissue oxygenation are associated with changes in the oxygen saturation of hemoglobin. Furthermore, these variations in oxygen concentrations indicate the hypoxic condition of the tumoral tissue. This manifests itself in low tissue oxygenation limiting normal cellular function and, consequently, increasing metabolic demand and angiogenesis [14, 21–23]. Such variations in oxygenation allow using optical imaging to determine the differences between tumors and healthy tissue, not only helping in diagnosis but also in monitoring patients’ response to therapies.

The variations in the concentrations of different absorbents emulating HbR and HbO could help in the characterization and calibration of tumor phantoms. In the case of breast tumors, it is therefore important to construct stable phantoms that allow the emulation the characteristic NIRS patterns of different degrees of malignancy of embedded tumors. However, phantoms made from actual blood constituents tend to naturally degrade. This behavior makes them impossible to reused over time, and a new one needs to be built for every calibration or experiment, making them relatively impractical [8, 14]. To overcome this problem, it is necessary to build phantoms with embedded tumor-like inclusions containing absorbing agents that reproduce the concentrations of the two hemoglobin species at the appropriate absorption wavelength in the near-infrared spectral range.

Generally speaking, an optical tissue phantom typically comes in one of three main physical forms: liquid, semisolid (agar-based matrix), or solid, each consisting of three fundamental building blocks, namely: a bulk material (water, epoxy resin, agar or agarose, silicone, etc.), a scattering agent (such as milk, Intralipid, acrylic paint, epoxy resin pigment, titanium dioxide or Lipovenos), and an absorbing agent (inks, toner, organic dyes or hemoglobin). The type of phantom to be developed must be chosen accordingly to the intended application. For example, liquid phantoms allow for a rapid and reproducible variation of their optical properties, but are, in general, not portable and may rapidly degrade depending on the bulk material [10, 12, 24–26]. On the other hand, solid phantoms, although their optical properties cannot be varied, are portable and stable, and are thus much more suitable for use as reference when testing different devices or reconstruction algorithms [27–32]. In particular, when emulating tumors, it is important that every phantom exhibits spectral absorption properties similar to HbO and HbR at their usual concentrations while also maintaining long-term optical stability to allow for repeated usage.

In this work, solid spheres made of epoxy resin that simulate tumors are presented, in which two different dyes are used to emulate the absorption properties of HbR and HbO in the optical window [29, 33, 34]. These spheres were made such to emulate both a malignant tumor, adenocarcinoma (ADC), and a benign tumor, fibroadenoma (FADN). The method presented here for fabricating solid optical tissue phantoms uses a common printer ink and a dye to control their absorption coefficient ($\mu_{a}$); these compounds are characterized by absorption behavior very similar to HbO and HbR, respectively, i.e., the printer ink absorbs light rather poorly near 830 nm but has its absorption maximum near 670 nm (thus mimicking the HbR behavior), while the dye molecules have a strong absorption peak near 830 nm but low absorption for λ≤ 670 nm, thus emulating the behavior of HbO. To achieve the desired reduced scattering coefficient ($\mu_{s}^{\prime }$), a titanium dioxide (${\text{TiO}}_{2}$)-based white pigment was used. Each phantom was optically characterized using Time Correlated Single Photon Counting (TCSPC) techniques [35, 36].

Finally, since the purpose of this study is to advance in the standardization of performance test methods for NIRS optical mammography, the phantoms were validated by analyzing continuous wave (CW) diffuse transmittance images and comparing them to Monte Carlo simulations. CW-NIRS techniques can be useful as a noninvasive tool with low cost and simplicity of operation. For a long time, this approach has demonstrated its versatility in detecting of changes in the concentrations of various chromophores present in biological tissues, especially under conditions that involve vascular compromise, such as the case of breast tumors, which are characterized by variations in oxyhemoglobin and deoxyhemoglobin [18, 37], as well for the study of metabolic changes in the brain [38–40].

This contribution is organized as follows: the next Section describes the materials and methods used to create the phantoms, including their optical characterization as well as the corresponding techniques used; Sect 2 is dedicated to presenting and discussing the diffuse transmittance imaging experiments performed to validate our phantoms. In Sect 3, the MC simulations of the imaging experiments are explained. The most important results are discussed in Sect 4. Finally, in Sect 5 the main conclusions reached are summarized.

As mentioned before, in this work we aim to design, construct, and test epoxy resin spheres to simulate a FADN and an ADC, to be inserted into a human breast-mimicking phantom. From the analyzed literature [14, 41–45] the range of values of the absorption coefficients reported for benign tumors (FADN type) and malignant tumors such as ADC vary for the different wavelengths. Taking these authors into account, it has been possible to establish that, for wavelengths around 670 nm, the $\mu_{a}$ range goes from 0.00253 ${\text{mm}}^{-1}$ to 0.013 ${\text{mm}}^{-1}$ for the FADN, and from 0.00962 ${\text{mm}}^{-1}$ to 0.05 ${\text{mm}}^{-1}$ for the ADC. Meanwhile, for 830 nm, the absorption coefficients vary between 0.0008 ${\text{mm}}^{-1}$ and 0.015 ${\text{mm}}^{-1}$ for the FADN and from 0.0008 ${\text{mm}}^{-1}$ to 0.024 ${\text{mm}}^{-1}$ for the ADC.

**Fig 1:**
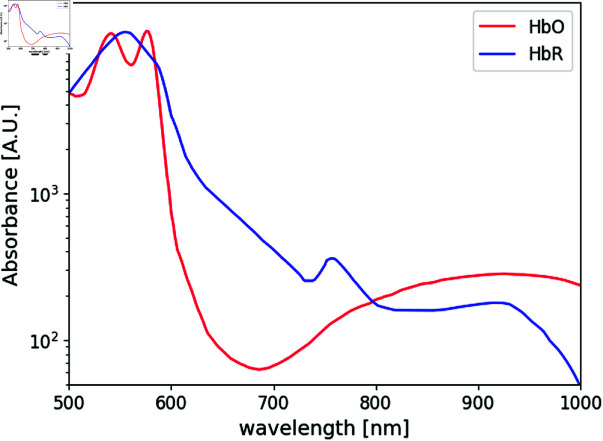
Absorbance spectra of oxy and deoxyhemoglobin in the range between 500 nm to 1000 nm [46].

Taking into account the spectral characteristics of HbO and HbR, shown in Fig 1, inclusions combining different concentrations of selected absorbing agents were designed to emulate the optical behavior of these two blood components when illuminated with NIR light.

Additionally, for the elaboration of the bulk, we tested two different types of materials: a liquid one, made of a mix of milk and water, which has the advantage of being easily modifiable, and a solid one made of silicone. This section thus discusses the method of construction and the characterization of both the spheres and the bulk, which will emulate the breast containing the tumors.

### 1.1 Construction of the inclusions

The fabrication of the resin spheres follows the guidelines given by [47]. Epoxy systems consist of two components: the epoxy resin itself and its curing agent. In this work, we use the PL-310/EL-237 system from Prodyser SA [48], in which the resin (Component A) must be mixed with half the weight of the curing agent (Component B). This results in a transparent, hard material, with a refractive index of 1.56 [29–31]. To achieve the desired optical properties, $\mu_{a}$ and $\mu_{s}^{\prime }$, absorbing and scattering agents must be added to the system.

**Fig 2:**
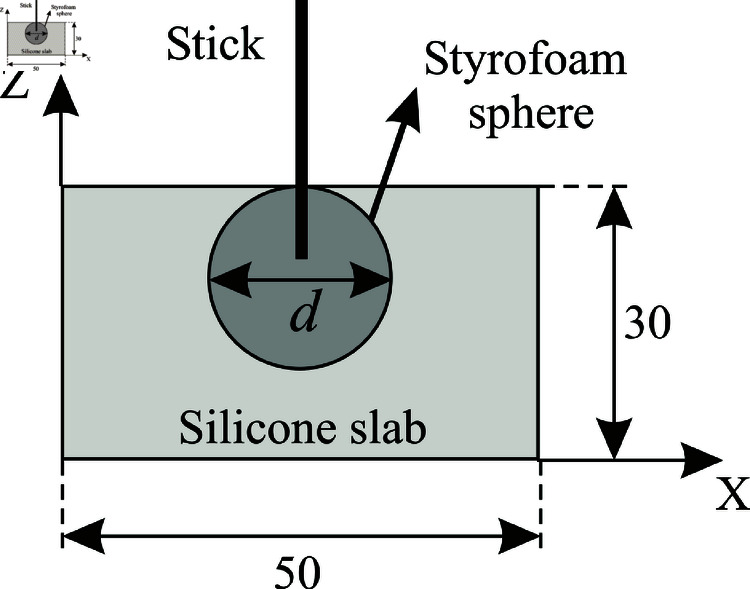
Schema of the silicone mold for constructing the inclusions. The stick serves both to hold the Styrofoam sphere in place and to extract it after the silicone has cured. The diameter *d* of the Styrofoam sphere is 10 mm or 20 mm, depending on the size of the resin inclusions being constructed. The cavity left behind is, in either case, filled with the desired resin mixture. The slab itself has dimensions (x,y,z) = (50 mm, 50 mm, 30 mm).

For constructing the spheres, we built different silicone casts, in which different resin mixtures were poured for curing. These casts were made by embedding a Styrofoam sphere (with the exact shape and size desired for the inclusion) in a slab which was then filled with liquid silicone covering the immersed sphere, as it is shown schematically in Fig 2. The sphere was placed just below the exposed surface of the silicone and thus, after the silicone had cured, the Styrofoam sphere was removed by simply pulling it out, leaving a spherical cavity behind. Then, and since silicone does not stick to resin, it is very easy to remove the spheres of cured resin from the silicone spherical mold. Two of these molds were constructed, the first one with a cavity of 10 mm in diameter and a second one with a cavity of 20 in diameter. This method was used to construct two types of spheres: homogeneous ones, corresponding to the smaller cavity, and core-shell structures, corresponding to the larger diameter. These concentric spheres were constructed by, first, making the 10 mm diameter resin sphere with the required absorption coefficient (the core) that is held centered inside the silicone mold with the 20 mm cavity. Second, by pouring the resin mixture with the corresponding absorption coefficient that characterizes the outer shell in the cavity that surrounds the smaller sphere.

**Fig 3:**
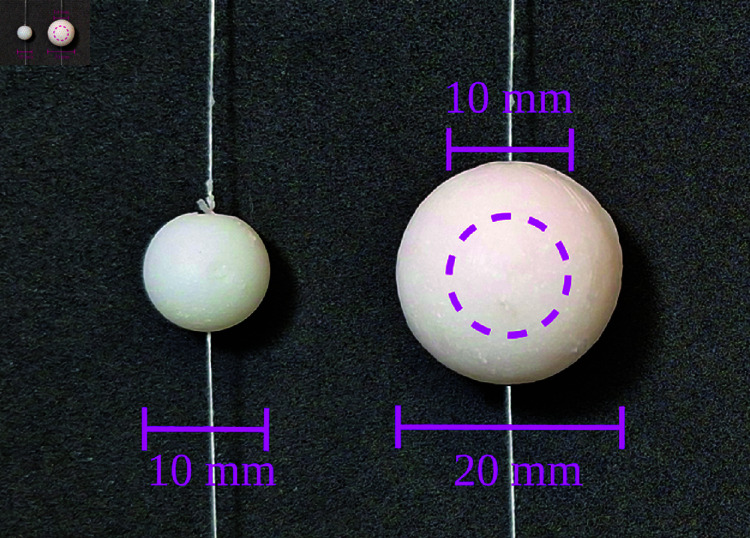
Photograph showing an example of the two types of resin inclusions constructed. The dashed pink circle represents the size of the core. The thin threads going through the solid spheres are used to hold them in place when they are added in the bulk phantom.

This method of constructing the inclusions, by combining different amounts of inks and dye in the inner and outer shells, is what allows to create ADC- or FADN-like inhomogeneities. A photograph of the inclusions is shown in Fig 3 and the corresponding proportions of dye and inks used to emulate an ADC or a FADN are listed in Table 2. Also, slab-type phantoms were constructed using the same resin mixture as each sphere, which were used to measure the optical properties of the emulated tumors.

**Table 1:** Molecular weights and molar extinction coefficients for both wavelengths, 670 nm and 830 nm, for the absorbents used in the fabrication of the inhomogeneities. (*) Since neither the exact composition nor the chemical formula are given by the manufacturer, the molecular weight was assumed equal to the Epson673 one.

	Molecular weight	ε (670 nm)	ε (830 nm)
Epson 673	68.14 g/mol	31409.55 g/mol cm	1520.56 g/mol cm
ADS830WS	827.49 g/mol	56049.20 g/mol cm	62906.73 g/mol cm
Powertec	(*)	—	—

As the scattering agent, we selected a white epoxy pigment concentrate (produced by Vacri, vacri.com.ar) that we used to emulate the diffusive nature of biological media, with the advantage of eliminating the sedimentation problems of other typical scattering agent like ${\text{TiO}}_{2}$ powder. As a first step, component A of the epoxy resin was mixed with the white epoxy pigment concentrate in a thermal bath at 50^∘^C to make the resin’s less viscous. The corresponding weight of component B was poured in a separate container and mixed with the appropriate absorbing agent emulating either oxy or deoxyhemoglobin. Then, parts A and B, with their respective amounts of scattering and absorbing agents, were mixed together for several minutes using a high-RPM mixer. A thorough mixing process is essential in order to achieve repeatability of the optical properties [49]. Finally, the entire mixture was poured into the selected mold and placed in a vacuum chamber for approximately five minutes to extract all air bubbles generated during the mixing.

As stated before, two different absorbents are needed to achieve differentiated absorption at λ=670 nm and λ=830 nm. We used a black printer ink, Epson 673 [50], to emulate the absorption of deoxyhemoglobin and ADS830WS [51], an organic dye manufactured by American Dye Source, Inc., to mimic the absorption of oxyhemoglobin [52]. All absorbance spectra were measured using a spectrometer (Shimadzu UV-1800), with a wavelength resolution of 1 nm working in the range of 400 nm to 1000 nm.

**Fig 4:**
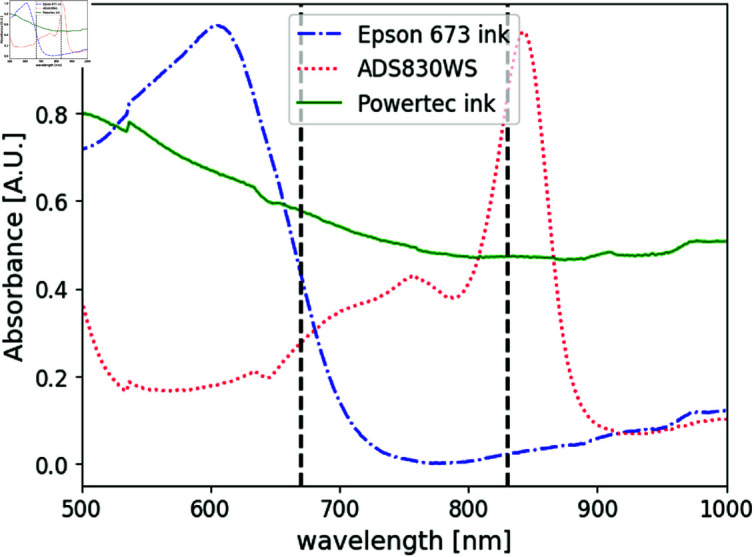
Absorbances of ADS830WS dye (red line), Epson 673 (blue line) and Powertec (green line) inks, in methanol and in resin. Black dot lines indicate the wavelengths employed in this work, 670 and 830 nm. It must be noted that the maximum absorption for Powertec ink is below 500 nm. Curves are normalized for better visualization of the absorption peaks.

The absorbance spectra of all three absorbers used are shown in Fig 4. The following procedure was employed to prepare the samples for the spectrophotometer: first, the dye or ink was dissolved in methanol. A small volume of this liquid was mixed with liquid resin and poured into a spectrophotometer cell. The cell was then placed in a vacuum chamber to remove air bubbles, and the mixture was allowed to solidify. The absorbance spectrum of the Epson 673 ink (blue line in Fig 4) shows a peak at ~ 600 nm and decreases gradually to negligible values at 830 nm; while for λ=670 nm (the wavelength of interest) the absorbance drops to about half its peak value. Moreover, because of the negligible absorption of this ink at 830 nm, a small fraction of a third ink, Powertec HP PIG 4844, which has been reported by the authors in a previous work [53], was also added. Accordingly to Fig 4 (green line), it presents an almost constant absorption for all wavelengths inside the region of interest [54]. Methanol was chosen as solvent for ADS830WS to correctly emulate the absorption of HbO at 830 nm. As the spheres were built in resin, in Fig 4 (red line) it can be seen the spectra for the dye solved in methanol, and already mixed with resin. It must be taken into account that ADS830WS dye need to be preserved in a dark place, since it is affected by bleaching. The same is true for the phantoms produced with this dye.

To replicate the optical properties reported in the literature mentioned previously in this Section, the concentrations of oxy and deoxyhemoglobin should be linked with the concentrations of the absorbents through their molar absorption coefficients $\varepsilon_{Dye}(\lambda_{1})$, $\varepsilon_{Dye}(\lambda_{2})$ and $\varepsilon_{Inks}(\lambda_{1})$, $\varepsilon_{Inks}(\lambda_{2})$, and solve Eq 1, which relates the $\mu_{a}$ with the concentrations of ink and dye that must be added.

$$\left[\begin{array}{l} \left[Dye\right]\\ \left[Inks\right] \end{array}]=[\begin{array}{ll} \varepsilon_{Dye}(\lambda_{1}) & \varepsilon_{Inks}(\lambda_{1})\\ \varepsilon_{Dye}(\lambda_{2}) & \varepsilon_{Inks}(\lambda_{2}) \end{array}]-1\begin{array}{l} \mu_{a}(\lambda_{1})\\ \mu_{a}(\lambda_{2}) \end{array}\right]$$ (1)

Once the concentrations, in μM (micromolar), of dye and ink have been obtained, the corresponding proportions in grams per liter of both are calculated with the molecular weights. In Table 1 the molecular weights of ADS830WS dye and Epson 673 ink are shown. The former is provided by the manufacturer [51] while the latter was calculated considering the composition of the ink [50].

**Table 2:** Concentrations of the absorbent materials, ADS830WS dye and Epson 673 and Powertec inks, for the tumor-like inclusions fabricated.

	Core			Shell		
	ADS830WS	Epson673	Powertec	ADS830WS	Epson673	Powertec
FADN	0	177,3 μM	134,8 μM	1,541 μM	0	0
ADC	0	1419,7 μM	134,8 μM	1,541 μM	0	0

### 1.2 Characterization of the absorbing materials

Now that the absorption of the selected absorbents to emulate the hemoglobin chromophores are known, it is interesting to obtain calibration curves to test how the absorption of phantoms behave with the addition of Epson 673 ink or ADS830WS dye. To this end, a set of curves of the absorption coefficient as a function of the concentration of the added absorbing agent was obtained for both, ADS830WS dye and Epson 673 ink. In accordance with the corresponding regions of the peak absorption shown in Fig 4, light at λ= 830 nm was used for the former, and at λ= 670 nm for the latter.

**Fig 5:**
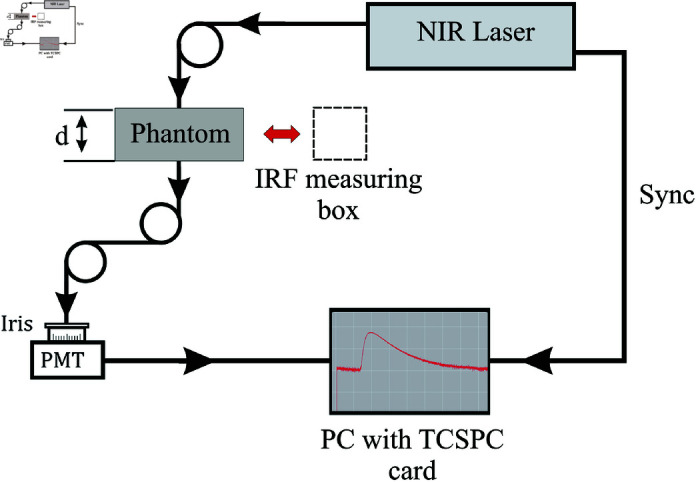
Experimental setup. Schematic representation of the experimental setup for measuring optical properties. To determine the Instrument Response Function (IRF), the phantom is replaced by a box of the same thickness containing neutral density filters and a diffuser to fill up all fibers modes.

The optical properties of resin phantoms were measured using a TSCPC (Time-Correlated Single Photon Counting) technique in a transmission slab geometry [55]. The experimental setup is shown in Fig 5. In this configuration, the slab was illuminated through a 600 μm diameter multimodal optical fiber (Thorlabs, NA = 0.39) connected, at the other end, to a semiconductor laser (Becker & Hickl, model BHLP-700) operating at λ= 670 nm or λ= 830 nm (depending on the absorbent to be measured), generating 70 ps pulses at a repetition rate of 50 MHz and with an average power of 0.3 mW and 0.4 mW respectively. At the opposite face of the slab, another fiber of 3 mm diameter (Dolan Jenner, USA, NA = 0.55) was placed aligned with the emission fiber to collect the diffusely transmitted light; and sent to a photomultiplier detector (PMT, Becker & Hickl PMC-100-20). Each time the PMT detects a photon, a pulse is sent to the photon counting board (Becker & Hickl SPC 130) which constructs a histogram of the number of detected photons as a function of the arrival time, thus building up the Distribution of Times of Flight (DTOF) of the photons. Finally, the resulting DTOFs were fed into a fitting routine, together with the Instrument Response Function (IRF) of the experiment to obtain the optical properties.

**Fig 6:**
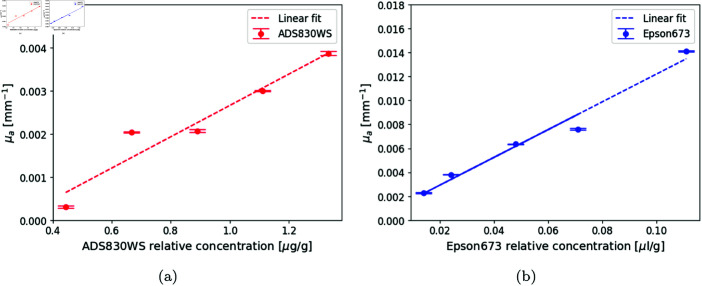
Variation of the absorption coefficient of a resin phantom. (a) against concentration of ADS830WS dye (in μg of dye per gram of resin), solved in methanol, at λ= 830 nm, and (b) as a function of concentration of Epson 673 ink, solved in methanol, at λ= 670 nm.

Five resin phantoms were built with different concentrations of ADS830WS, and another five with different concentrations of Epson 673, while the added amounts of the scattering agent remained constant. In the case of the dye, it is important to note in its absorbance curve, Fig 4, that at λ=670 nm it is not zero. This absorption triggers fluorescence, at around 700 nm. To overcome this problem, a long pass filter (Thorlabs FESH0700) was placed in front of the photo-multiplier. This setup allowed us to measure only the DTOFs affected by scattering and absorption effects, corresponding to λ= 830 nm. Fig 6a shows the measured $\mu_{a}$ for ADS830WS, and Fig 6b for the Epson 673 ink.

For both absorbents, the concentration was varied progressively and the expected increase in the absorption coefficient was observed.

To assess the temporal stability of the resin phantoms’ optical properties, reduced scattering and absorption coefficients were measured over a five-month period following fabrication. No significant variations were observed in these parameters. Specifically, the absorption coefficient was determined at 830 nm for phantoms with ADS830WS dye concentrations ranging from 0.44 μg/g to 1.1 μg/g. Over the period of 150 days post-fabrication, a 3% decrease was observed in most phantoms. However, the phantom with 0.67 μg/g of ADS830WS dye exhibited an 18% decrease, likely due to prolonged light exposure. Similarly, absorption coefficients of phantoms with varying Epson 673 ink concentrations were measured at 670 nm from fabrication day, until 150 days after. The temporal variation in absorption observed in these phantoms was around 7%.

### 1.3 Construction of the bulk phantom

For the transmittance experiments detailed in the next section, a host medium is needed to embed the inclusions in either liquid or solid phantoms that simulate the surrounding (normal) breast tissue. To prepare the liquid phantom, we made a mixture of whole milk (Ilolay, 3% fat) and distilled water. No absorbent was used for these experiments in particular. This mixture was poured into a 40 mm thick glass cuvette, where the spherical resin inclusion was placed, at *z* = 20 mm. The solid phantom was made of silicone and was manufactured following the lines detailed in [56]. We selected a platinum-catalyzed silicone rubber, Silcast 815 (Novarchem, Argentina) that consists in two components, the silicone itself and the curing agent, which are to be mixed in equal quantities. As scatterer we used white acrylic paint (Monitor, number 801 “Titanium White”, Argentina) [56], while Powertec ink was used as the absorbent, since the absorption coefficient of these phantoms when no ink is added tends to zero. Additionally, 10% of silicone fluid was incorporated in to decrease the hardness of the resulting phantom. With the described mixture, we built a 40 mm thick slab phantom with the inclusion located at its center in depth, in the same fashion as in the liquid phantom case. All bulk phantoms were optically characterized using the same TCSPC technique used for the inclusion phantoms.

## 2 Diffuse transmittance imaging experiments

**Fig 7:**
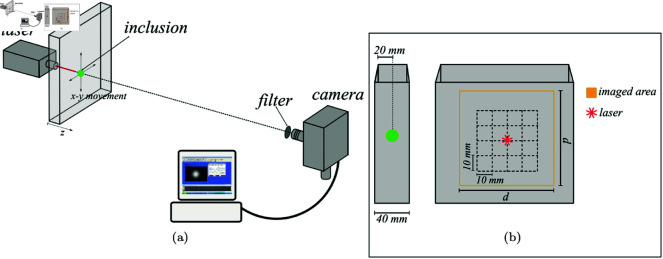
Experiment schematics. (a) showing the disposition of source, detector and phantom, and (b) of the grid followed by the inclusion in its movement, the photographed area and the position on the inclusion in the *z* axis.

In order to test the ability of the proposed phantoms to mimic the patterns of HbR and HbO that we expect to see when measuring real lesions using NIRS imaging techniques, transmittance experiments with two wavelengths, 670 nm and 830 nm were performed. Though the light sources were the same pulsed lasers mentioned in Subsect 1.2, due to their high repetition rates (50 MHz) and the relatively long integration times to acquire the images, ≈0.5 *s*, they can be considered as continuous wave sources. The detector was an EMCCD camera (Andor iXon Ultra 897), focused on the side of the cuvette (or the silicone slab) opposite to the illumination, and centered at the optical axis determined by the collimated laser beam. In Fig 7a we present a schematic drawing of the experiment, where it can be seen the laser, as it illuminates the phantom, and the camera. Fig 7b shows the location of the inclusion in depth (left) and the imaged area registered by the CCD detector (right) together with a grid indicating the different in plane positions of the inclusion for each photo, covering an area of 40 mm × 40 mm in 10 mm steps. Thus 25 images were acquired for the normalization process described in Ref. [57].

The same configuration shown in Fig 7 for the liquid phantom was used for the solid silicone phantom. In both cases, the camera lens was adjusted such as to obtain an image size of 80 mm × 80 mm (*d* × *p* in Fig 7b). As mentioned in Sect 1.2, a filter must be used when working with λ=670 nm to avoid fluorescence from the ADS830WS dye contained in the inclusions.

## 3 Monte Carlo simulations

**Fig 8:**
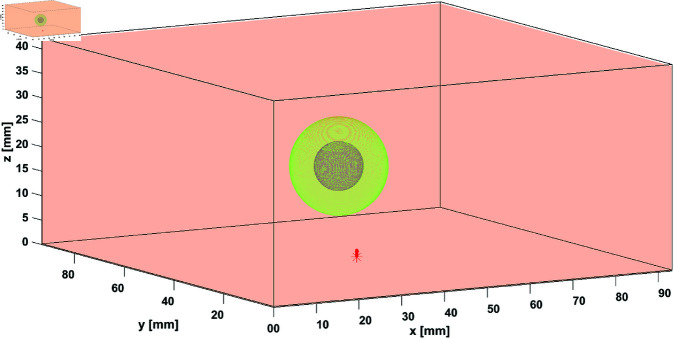
MC digital phantom. 3D representation of the digital phantom used for the MC simulations containing the core-shell inclusion emulating either an ADC or a FADN. The red arrow at the bottom represents the source location and the illumination direction.

Monte Carlo simulations represent the gold standard method for simulating light propagation in turbid and semitransparent media [58] as well as in other physics areas [59]. In this work, we used MC simulations, for which we utilized the MCX program [60, 61], to validate the constructed phantoms. To this end, we designed a volume of $200\times 200\times 40\;{\text{mm}}^{3}$ as the bulk medium, with different types of spherical inclusions embedded in it (Fig 8). In particular, the two inclusions form a structure, having an inner core that emulates the behaviour of HbR, surrounded by a shell emulating the HbO.

A pencil beam source was directed to the geometrical center of the x-y plane (the entrance face of the medium), and the photons were detected at the opposite face, thus emulating a CW transmittance experiment, with the opposite face of the phantom acting as the camera.

Each inclusion was moved inside a 40 mm × 40 mm regular grid, with a step length of 10 mm, thus yielding a total of 25 inclusion locations, in order to replicate the experiment; for each position of the inclusion $1\times 10^{9}$ photons were launched. Each set of 25 simulations was run twice: the first one with the absorption and scattering coefficients corresponding to λ = 670 nm and the second one with the absorption and scattering coefficients corresponding to λ = 830 nm. The optical characteristics of all the media involved in the simulations are shown in Table 3, and the geometrical details are given in the following section.

**Table 3:** Optical properties of the tumor-like inclusions and the hosts.

	λ=670 nm		λ=830 nm	
	$\mu_{a}\;[{\text{mm}}^{-1}]$	$\mu_{s}^{\prime }\;[{\text{mm}}^{-1}]$	$\mu_{a}\;[{\text{mm}}^{-1}]$	$\mu_{s}^{\prime }\;[{\text{mm}}^{-1}]$
FADN Core	0.00451	0.919	0.00102	0.731
ADC Core	0.0179	0.883	0.000972	0.760
FADN & ADC Shell	0.00210	1.158	0.00265	0.821
Liquid Host	0.000811	1.095	0.00209	0.759
Solid Host	0.00177	0.954	0.00125	0.772

Each one of the 50 (GPU-parallelized) simulations took about 300 seconds to complete running on a workstation equipped with a Nvidia Titan Xp GPU, a Ryzen 5700X CPU and 64 GB of RAM. That is, a total run time of about 17 hours.

The processing of the obtained images was carried out using the image processing toolbox of Matlab software; it consists of:

- Averaging the 25 images obtained for each wavelength.
- Using the average obtained in the previous step to normalize the image that correspond to the inclusions located at the center.
- Computing the mean total pathlengths (MTPLs) of photons in the turbid media and using them to go from the normalized image (attenuation) to a map of changes in $\Delta \mu_{a}$, by means of the Modified Beer-Lambert law (MBLL):
  $$\Delta A(\lambda )=-\ln\left(\frac{I(\lambda ,\rho )}{I_{0}(\lambda )}\right)=L\cdot \Delta \mu_{a},$$ (2)
  where ΔA(λ) represents the change in attenuation (for each wavelength) and *L* is the MTPL of photons, that were obtained by implementing Eq (47) from [35]. $\Delta \mu_{a}$ is the change in the absorption with respect to the background ($\mu_{a,\text{bulk}}$), and it is originated by the changes in the concentrations of both the dye and the ink. That is:
  $$\Delta \mu_{a}(\lambda )=\varepsilon (\lambda )\cdot \Delta C,$$ (3)
  where
  $$\varepsilon =[\begin{array}{cc} \varepsilon_{Dye}(\lambda_{1}) & \varepsilon_{Inks}(\lambda_{1})\\ \varepsilon_{Dye}(\lambda_{2}) & \varepsilon_{Inks}(\lambda_{2}) \end{array}],$$ (4)
  and
  $$\Delta C=[\begin{array}{c} \Delta [Dye]\\ \Delta [Inks] \end{array}].$$ (5)
- Using the equation given in (1).

## 4 Results and discussion

The required concentrations of both inks and dye, used in the inclusions were estimated using Eq 1. As mentioned, the cores of the inclusions are solid spheres, with different proportions of inks, that emulate the absorption corresponding to a FADN and an ADC respectively. On the other hand, the external shell was prepared with the same proportion of ADS830WS dye for both inclusions. The corresponding concentrations for each inclusion are shown in Table 2.

The optical properties obtained for the inner sphere (i.e. the core) and the shell of the inclusions at both wavelengths of interest, as well as those of the silicone and liquid hosts, are shown in Table 3. Although the values obtained are within the range of reported values, they are lower and more demanding in terms of variations with respect to the host medium. However, the absorption coefficient in the core of the ADC is approximately four times that of the FADN, simulating a higher concentration of HbR in the malignant tumor.

The increase in the absorption coefficient of the liquid host (for which milk is used as scattering agent) at λ=830 nm compared to λ=670 nm, can be attributed to the fact that the scattering particles in the milk are mostly fat, whose absorption increases with λ [62].

### 4.1 CW transmittance experiments

Transmittance experiments were performed using both the liquid and the solid host and for each of the inclusions (ADC and FADN) immersed in them one at a time. Each experiment was repeated for λ=670 nm and λ=830 nm.

The obtained results are shown as plots of the normalized transmitted intensity according to the normalization process mentioned in Sect 2 and detailed in Ref. [57]. The images shown in each subplot correspond to the experimental situation in which the sphere was placed in the position closest to the axis formed by the laser illumination point and the optical axis of the camera lens, at a depth equal to half the host thickness (some deviation is expected since there is no way to exactly know the precise position of the inclusion relative to the host edges). The corresponding normalized intensity profiles along the center of the inclusion (indicated with a black dashed line) are also shown for the sake of clarity. At this point, and taking into account that far from the inclusion, the normalized intensity tends to unity, it is useful to define the contrast, C=|1−M|, where *M* is the minimum or maximum normalized intensity, to compare the results obtained later on. The resulting contrasts are summarized in Table 4.

**Table 4:** Summary of the contrasts obtained from the normalized images for both inclusions as a function of the host type and the wavelength.

	Host	λ=670 nm	λ=830 nm
FADN	liquid	0,35	0,21
	solid	0,29	0,24
ADC	liquid	0,5	0,28
	solid	0,41	0,27

**Liquid Host Phantom**

**Fig 9:**
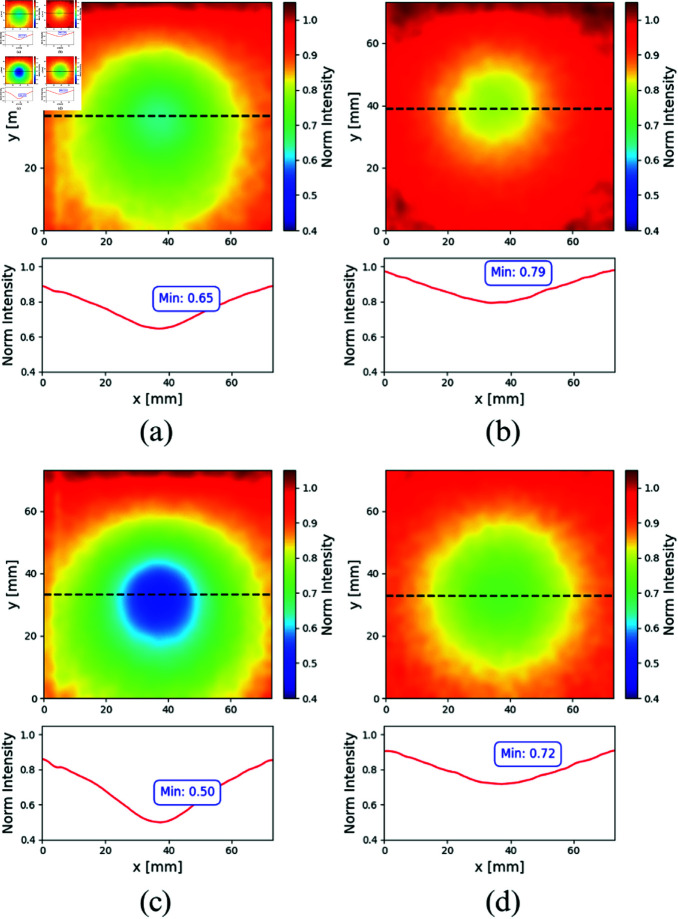
Normalized transmittance images and corresponding horizontal profiles for the inclusion emulating the FADN (upper row) and for that emulating the ADC (bottom row), immersed in a liquid host. The left column corresponds to λ=670 nm while the right column to λ=830 nm. The black dashed lines in the images display where the profile was taken.

Fig 9 shows the corresponding constructed images for core-shell inclusions immersed in the liquid host: FADN (Fig 9a and 9b) and ADC (Fig 9c and 9d), and their corresponding horizontal profiles. From themse images, it can be observed that for both inclusions, when illuminated with λ=830 nm (right column), the results are similar, i.e. the drop in the normalized intensity reaches quite similar values, with C=0,21 for the FADN and C=0,28 for the ADC. This is consistent with the fact that both inclusions have the same concentration of dye in their shells.

On the other hand, at λ=670 nm, the FADN, Fig 9a is easily distinguishable from the ADC (Fig 9c), due to the difference in Epson 673 ink concentration. For the FADN, the obtained contrast is C=0,35, while for the ADC, it is C=0,50. The same trend can be observed in their corresponding profiles.

**Solid Host Phantom**

**Fig 10:**
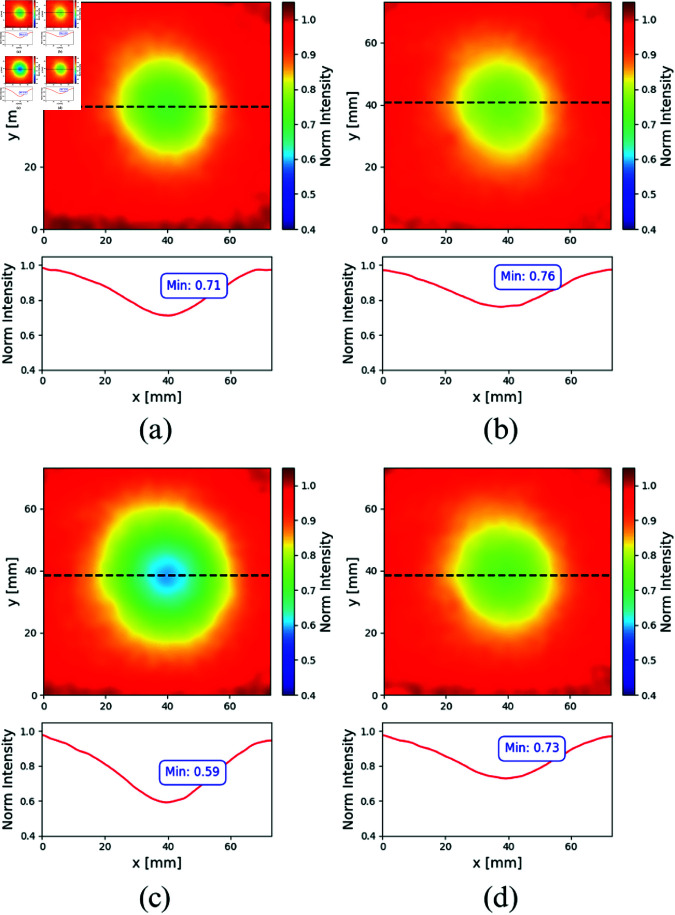
Normalized transmittance images and corresponding horizontal profiles for the inclusion emulating the FADN (upper row) and the ADC (bottom row), immersed in a solid silicone host. Left column corresponds to λ=670 nm, right column to λ=830 nm.

A similar set of experimental results is shown in Fig 10, but in this case, for both inclusions immersed in the silicone slab, the optical properties of which are summarized in Table 3.

The subplots (a) and (b) in Fig 10 correspond to the FADN, while the results for the ADC-like inclusion are shown in Fig 10c and 10d. At λ=670 nm, the contrast for the FADN, C=0,29, is lower than the contrast for the ADC, C=0,41, which is consistent with the increase in the concentration of Epson 673 ink.

On the other hand, for λ=830 nm, the contrasts are similar for both inclusions (as it happened with the liquid bulk), being *C* = 0.24 for the FADN and *C* = 0.27 for the ADC. The difference in the depth of the profiles (and thus the corresponding contrasts) between both inclusions when comparing the milk and the silicone hosts, is related to the variations in the optical properties of these bulk media.

In the following Table 4 the contrasts obtained for the experiments presented are summarized.

### 4.2 Comparison with Monte Carlo simulations

**Fig 11:**
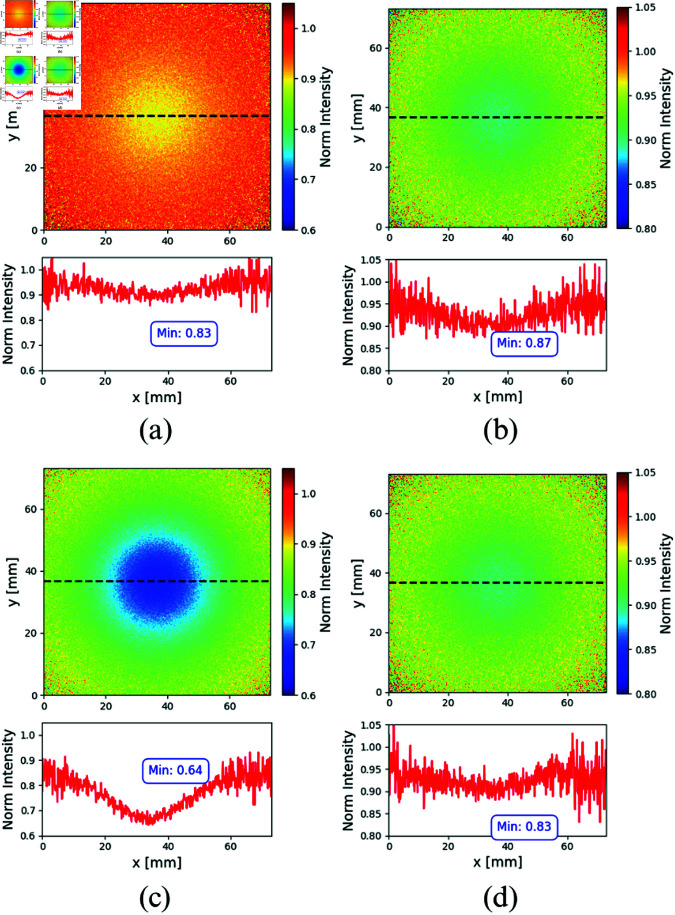
MC results. Reconstructed Monte Carlo transmittance images and horizontal profiles for the inclusion emulating the FADN (upper row) and for that emulating the ADC (bottom row) in the silicone host. The left column corresponds to λ=670 nm while the right column to λ=830 nm.

As a test for our method we compared the resulting experimental normalized images with those obtained by MC simulations. To this end, the *structural similarity index measure* (SSIM) was computed. This measure ranges from 0 —completely different images—, to 1 —identical images— [63, 64]. To obtain the SSIM, an area of 128 × 128 pixels, centered in the position of each inclusion (coincident with the illumination point) was cropped from the MC and for the experimental images of both, ADC and FADN scenarios; the reason of doing so is to discard the pixels that are rarely reached by photons, that would greatly increase the similarity between images but carry no information. Furthermore, the MC data was processed with a median filter to reduce the pixel-to-pixel noise typical of MC simulations. Without this step, the SSIM comparison would be dominated by the differing characteristics of the background noise, resulting in an artificially low similarity score. The strength of the median filter was set to 7 pixels, a value estimated based on the average free path of photons in the studied medium. This correlates with the minimum characteristic size that can be reliably retrieved from the transmittance images. The SSIM was computed using the corresponding built-in function of Matlab. Fig 11 shows the normalized images resulting from MC simulations for the case of FADN (upper line) and for ADC (lower line), and for both wavelengths used. For brevity, MC simulations are shown only for the solid silicone host; results obtained for the liquid milk host lead to similar conclusions.

For the case of the ADC images, the SSIMs are 0.7916 for the images at λ=670 nm and 0.7184 for the ones λ=830 nm. In the case of the FADN images, the SSIMs are 0.7574 for λ=670 nm and 0.7181 for the λ=830 nm. While the observed SSIM values may appear relatively low, it is crucial to consider that we are comparing data originating from fundamentally different sources. Specifically, the noise characteristics—such as signal-to-noise ratio (SNR) and noise distribution—differ between the Monte Carlo simulations and experimental data. Despite our use of a median filter to reduce noise, these inherent differences affect the similarity between the images. For brevity, MC simulations are shown only for the solid silicone host; results obtained for the liquid milk host lead to similar conclusions.

### 4.3 Retrieval of concentration maps of the absorbents

**Fig 12:**
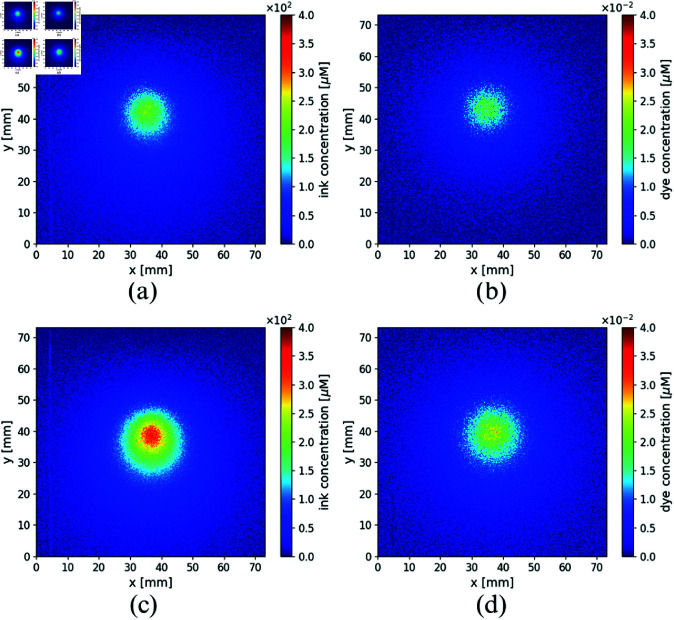
Concentrations maps of inks (left column) and dye (right column) obtained from the experiments, for both inclusions: (a) and (b) FADN, (c) and (d) ADC, immerse in the liquid milk host, corresponding to the experimental results in Fig 9.

**Fig 13:**
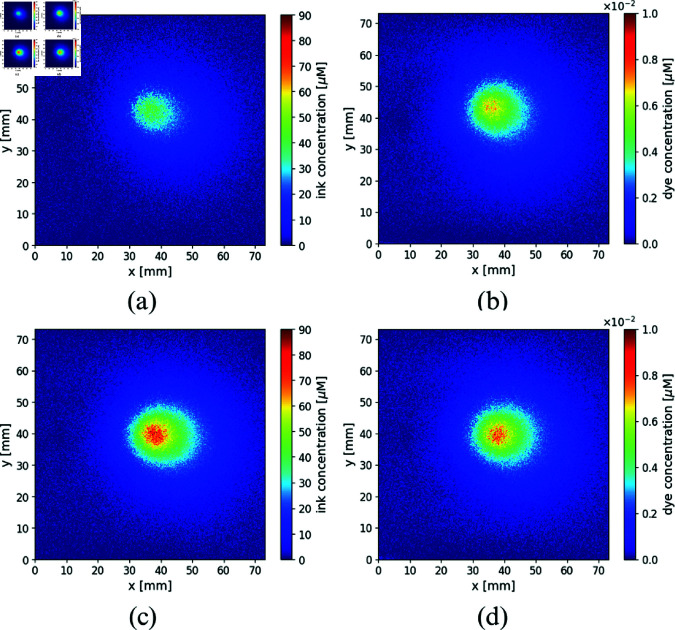
Concentrations maps of inks (left column) and dye (right column) obtained from the experiments, for both inclusions: (a) and (b) FADN, (c) and (d) ADC, immerse in the silicone host, corresponding to the experimental results in Fig 10.

Using the MBBL (see Eq 2) and the experimental images of the normalized intensities described in Section 3, the concentrations of the absorbents (inks and dye) present in the immersed inclusions could be obtained. Note that since, as said, images are normalized to the background, and because measurements were done in CW, only concentrations relative to this normalization background are retrieved. Fig 12 shows the concentration maps of ink and ADS for the FADN and the ADC, when using the liquid host phantom. In a similar way, Fig 13 represents the concentration maps of ink and ADS for the fibroadenoma and the adenocarcinoma, for the silicone host phantom.

Both figures show a higher concentration of inks in the ADC compared to the FADN. In particular, when the host medium is milk, the inks concentration in the ADC is twice than in the FADN, and in silicone host the ratio is greater, of approximately three times for the inclusion that simulates the malignant tumor. Regarding dye concentrations (ADS), the relative values recovered are very similar in both inclusions and in both host media. Even if the absolute retrieved values do not match the nominal concentrations used in the construction of the lesions, this can be understood considering that experiments, and thus the concentration retrievals based on them, were performed in CW; thus the imaged intensity obtained for each wavelength carries information about an integrated volume containing the host and two layers of the respective inclusions. The relative qualitative concentrations obtained are, however, fairly good and reproduce quite well the original situation.

## 5 Conclusions

In this paper we have successfully constructed and tested tumor-like inclusions emulating both FADN and ADC. They were made using combinations of an ink (Epson 673) and a dye (ADS830WS) which, together, reproduce the variations in HbO and HbR that are related to the metabolic characteristics of these types of breast tumors.

We have shown that they can not only be constructed as a core-shell structure (like these tumors are structured), but they can also be properly differentiated by imaging the medium from the outside through the MBBL, which allows the retrieval of the concentrations of both substances that emulate both hemoglobin species. According to SSIM calculations, the normalized transmittance images from the experiment agree fairly well with the corresponding Monte Carlo simulations.

Since this type of phantom, particularly the silicone containing resin inclusions, is also durable and does not decompose with time, it is suitable for calibrate and adjust optical mammography instruments, varying the relative concentrations of the two absorbents, to compare the performance of different devices. Although a reasonable qualitative behavior has been obtained in terms of the relative concentrations of inks and dye with respect to the host, additional work using time resolved techniques in tomographic configurations is foreseen for a more detailed study of this type of breast phantoms.
